# One-Step Preparation of Large Area Films of Oriented MoS_2_ Nanoparticles on Multilayer Graphene and Its Electrocatalytic Activity for Hydrogen Evolution

**DOI:** 10.3390/ma11010168

**Published:** 2018-01-22

**Authors:** Jinbao He, Cristina Fernández, Ana Primo, Hermenegildo Garcia

**Affiliations:** Instituto Universitario de Tecnología Química CSIC-UPV, Universitat Politecnica de Valencia, Av. de los Naranjos s/n, 46022 Valencia, Spain; jinhe@itq.upv.es (J.H.)

**Keywords:** molybdenum disulfide, oriented nanoparticles, multilayer graphene films, hydrogen evolution, electrocatalyst

## Abstract

MoS_2_ is a promising material to replace Pt-based catalysts for the hydrogen evolution reaction (HER), due to its excellent stability and high activity. In this work, MoS_2_ nanoparticles supported on graphitic carbon (about 20 nm) with a preferential 002 facet orientation have been prepared by pyrolysis of alginic acid films on quartz containing adsorbed (NH_4_)_2_MoS_4_ at 900 °C under Ar atmosphere. Although some variation of the electrocatalytic activity has been observed from batch to batch, the MoS_2_ sample exhibited activity for HER (a potential onset between 0.2 and 0.3 V vs. SCE), depending on the concentrations of (NH_4_)_2_MoS_4_ precursor used in the preparation process. The loading and particle size of MoS_2_, which correlate with the amount of exposed active sites in the sample, are the main factors influencing the electrocatalytic activity.

## 1. Introduction

Molybdenum disulfide (MoS_2_) is among the most efficient non-noble metal electrocatalysts for water oxidation and reduction, and it has been proposed as an economically-viable alternative to the use of platinum in electrocatalysis for water splitting [1,2,3]. MoS_2_ is a 2D layered material that upon exfoliation can be supported on graphene (G), and in this way, electrodes with high performance for hydrogen generation from water have been developed [4,5]. It has been found that the electrocatalytic activity for the hydrogen evolution reaction of MoS_2_ increases as the particles become thinner and with the presence of steps, edges and defects on the particles [4,5].

Recently, we have reported an innovative method for the preparation of hybrid MoS_2_/G materials as powders [6]. The method is based on the simultaneous formation of G and MoS_2_ by pyrolysis at temperatures above 900 °C of a mixture of alginate containing (NH_4_)_2_MoS_4_. It was previously known that the pyrolysis of certain natural polysaccharides such as alginate and chitosan gives rise to the formation of turbostratic graphitic carbon residues that can be easily exfoliated with high efficiency to form G suspensions [7]. Since these polysaccharides are good adsorbents, if the biopolymer contains metal ions or some other component, spontaneous phase segregation during the pyrolysis may lead to the formation of Gs having metal nanoparticles (NPs) or some other metal compound present on the G surface [8,9,10]. This was the case of powders of alginate containing (NH_4_)_2_MoS_4_ that upon pyrolysis and subsequent exfoliation of the carbon residue led to the formation of MoS_2_ platelets deposited on G [6]. The simultaneous formation of MoS_2_ and G in the process was assessed by XRD and Raman spectroscopy of the powders, recording the characteristic signature of both materials and also by transmission electron microscopy (TEM) and atomic force microscope (AFM) images of the resulting particles in suspension upon sonication of the powders [6]. The MoS_2_/G material suspended in water was used as the catalyst for the visible light hydrogen generation using Eosin Y as the photosensitizer and methanol as the sacrificial electron donor [6]. 

Besides as powders, some of these natural polysaccharides such as alginates and chitosan are able to form defectless films of nanometric thickness and subnanometric rugosity that upon pyrolysis are converted into films of G or few-layer G [11]. The ability of some of these natural biopolymers to form films of nanometric thickness is directly responsible for the generation of the corresponding single or few-layer G films upon graphitization. When the film of polysaccharide contains some adsorbed metal ion that does not form metal carbides, such as Au, Pt and Cu, their pyrolysis leads to the formation of G films having strongly anchored metal NPs exposing in several cases some preferential facet orientation [8,9,10,12]. Continuing with this line of research, it would be of interest to exploit further this innovative preparation procedure of films based on the pyrolysis of precursor polysaccharides for the one-step preparation of films of MoS_2_ supported on few-layer G that can be used directly as electrodes and measure their activity as electrocatalysts for hydrogen generation from water. 

This type of electrocatalytic measurement is described in the present paper, by preparing large area films (2 × 2 cm^2^) of MoS_2_ supported on G by pyrolysis of films of ammonium alginate containing (NH_4_)_2_MoS_4_. It was found that as a consequence of the preparation procedure, the resulting MoS_2_ particles exhibit a preferential 002 facet orientation and present electrocatalytic activity for H_2_ evolution without the need for an additional conductive electrode.

## 2. Results and Discussion

### 2.1. Sample Preparation and Characterization

**Figure 1:**
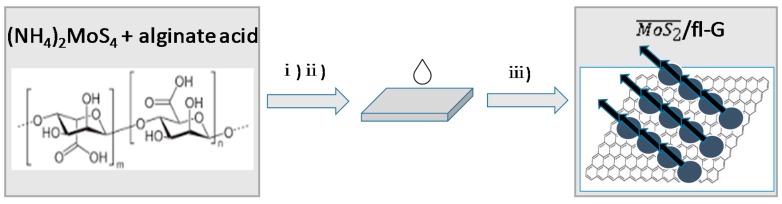
Preparation procedure for $\bar{{\mathrm{MoS}}_{2}}$ml-G (G, graphene; ml, multilayer) films supported on quartz: (i) filtration of the aqueous solution of ammonium alginate containing (NH_4_)_2_MoS_4_; (ii) spin coating of alginate solution on clean quartz; (iii) pyrolysis at 900 °C under Ar atmosphere.

Figure 1 illustrates the preparation procedure of $\bar{{\mathrm{MoS}}_{2}}$/ml-G ($\bar{{\mathrm{MoS}}_{2}}$ meaning oriented MoS_2_, ml meaning multilayer). The process starts by dissolving alginic acid in an aqueous solution with the aid of ammonia. To this alginate solution, the required amount of (NH_4_)_2_MoS_4_ was added. These solutions containing (NH_4_)_2_MoS_4_ and ammonium alginate were spin cast on clean glass substrate, and the resulting ammonium alginate film was pyrolyzed under Ar at 900 °C. Several films, where the loading of (NH_4_)_2_MoS_4_ was varied by using initial (NH_4_)_2_MoS_4_ concentrations from 1–60 mM, were used. This resulted in a set of $\bar{{\mathrm{MoS}}_{2}}$/ml-G-χ, where the χ value denotes the initial (NH_4_)_2_MoS_4_ concentration used in the preparation of these films.

**Figure 2:**
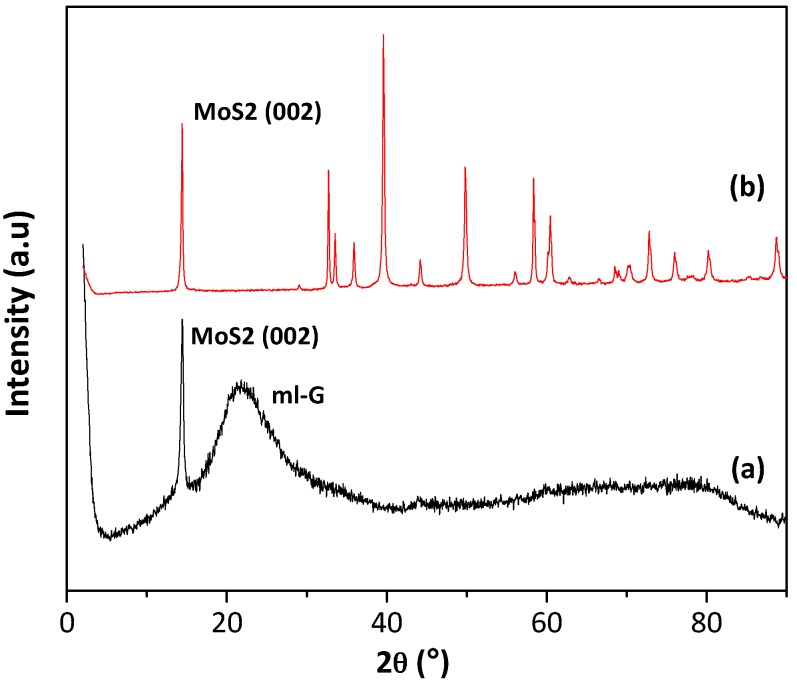
XRD patterns of $\bar{{\mathrm{MoS}}_{2}}$/ml-G-60 film (**a**) and the commercial MoS_2_ powder (**b**). Note that the broad band of about 27° in Plot a is due to ml-G.

The films resulting after the pyrolysis were initially characterized by XRD. As expected in view of related precedents, only the samples prepared with the highest (NH_4_)_2_MoS_4_ concentrations showed some XRD peaks characteristic of MoS_2_. Figure 2 shows the XRD pattern recorded for commercial MoS_2_ powder and for a sample prepared using (NH_4_)_2_MoS_4_ as the precursor with a concentration of 60 mM, where in addition to a broad band corresponding to graphitic carbon (multilayer G) of about 24°, a reflection at 14° corresponding to the 002 diffraction of MoS_2_ could also be recorded. No other diffraction peaks expected for MoS_2_ were present. The absence of the peaks corresponding to other diffraction planes indicates that MoS_2_ particles are formed exhibiting a preferential 002 facet orientation. Earlier precedents have shown that pyrolysis of thin films of alginate and chitosan containing Au, Pt, Ag and Cu among other metals can result in metal nanoplatelets supported on G exhibiting preferential 111 facet orientation [8,10,12]. The present case will constitute an additional example of the formation of MoS_2_ with the preferential exposure of the 002 facet. In accordance with earlier proposal, the most likely reasons for this preferential particle growth are the template effect of graphene layer on the nascent MoS_2_ nanoparticle and/or the higher thermodynamic stability of the 002 surface against other crystal facets.

**Figure 3:**
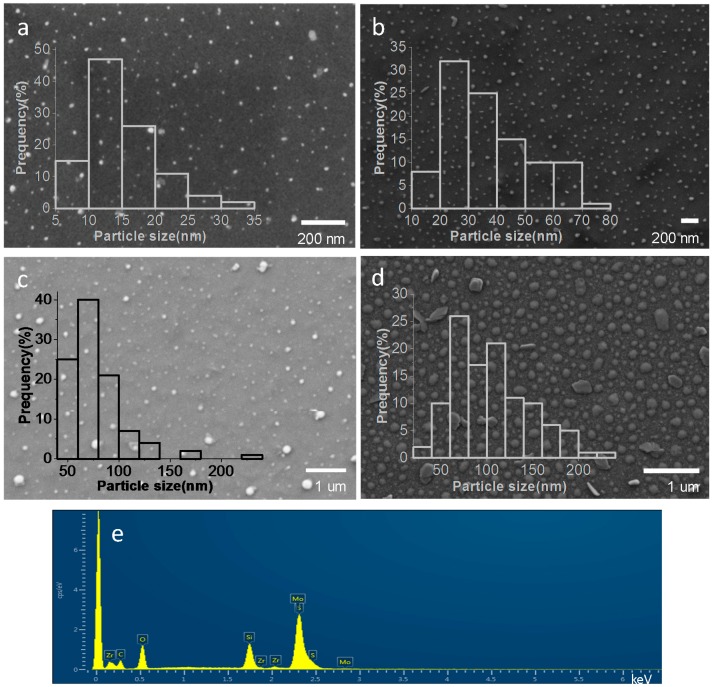
Representative SEM images of $\bar{{\mathrm{MoS}}_{2}}$/ml-G-0.5 (**a**), $\bar{{\mathrm{MoS}}_{2}}$/ml-G-2 (**b**), $\bar{{\mathrm{MoS}}_{2}}$/ml-G-5 (**c**), $\bar{{\mathrm{MoS}}_{2}}$/ml-G-10 (**d**), as well as the different histograms of MoS_2_ particle size distribution and the EDS analysis result of the $\bar{{\mathrm{MoS}}_{2}}$/ml-G-2 (**e**) confirming that the particles correspond to MoS_2_ supported on ml-G. Note that the presence of Zr and Si is due to the sample holder used for SEM and the quartz substrate, respectively.

The morphology of MoS_2_ nanoplatelets and their average particle size on the samples were determined by scanning electron microscopy (SEM). Figure 3 presents the images of the films prepared with initial (NH_4_)_2_MoS_4_ concentrations of 0.5 mM, 2 mM, 5 mM and 10 mM, showing that the $\bar{{\mathrm{MoS}}_{2}}$/ml-G samples contain particles of MoS_2_ distributed all over the G film with a broad particle size distribution ranging approximately from 10–200 nm. Quantitative analysis of the particles observed in the SEM images by energy dispersive X-ray spectroscopy (EDS) confirmed that these particles are constituted by Mo and S in an approximate atomic 1:2 ratio, providing firm evidence of the composition of the particles seen in the images. The most important conclusion of this SEM study is that there was a clear relationship between the average MoS_2_ particle size and the concentration of (NH_4_)_2_MoS_4_ used in the preparation, the average particle size increasing along the concentration of (NH_4_)_2_MoS_4_. In this way, the $\bar{{\mathrm{MoS}}_{2}}$/ml-G-x with the smallest average particle size of 15 nm corresponded to $\bar{{\mathrm{MoS}}_{2}}$/ml-G-0.5, while the average particle size grows to 37, 78 and 105 nm for $\bar{{\mathrm{MoS}}_{2}}$/ml-G-2, $\bar{{\mathrm{MoS}}_{2}}$/ml-G-5 and $\bar{{\mathrm{MoS}}_{2}}$/ml-G-10, respectively. It is known that an increase in particle size has a detrimental influence on the electrocatalytic performance that decreases as the particles become larger. 

**Figure 4:**
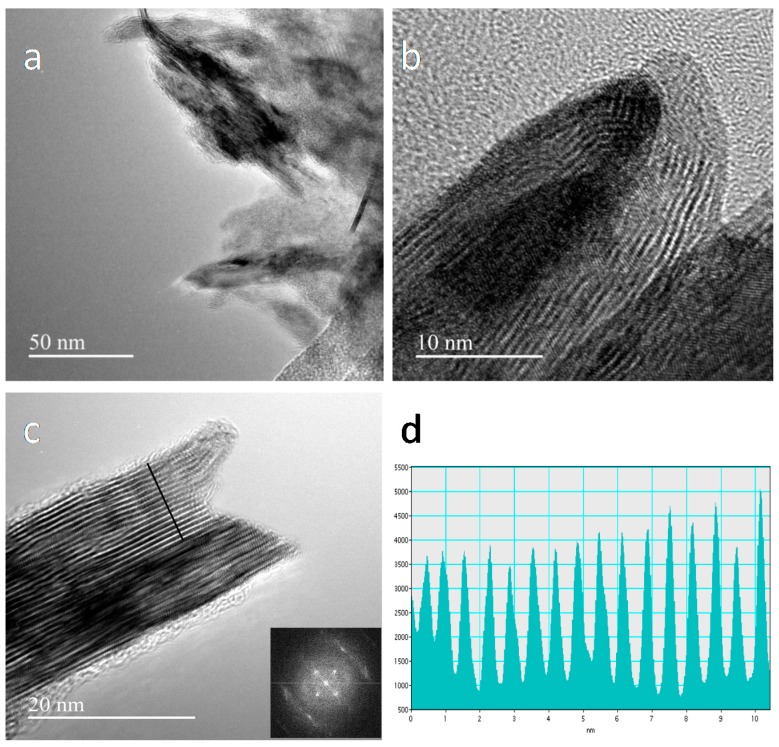
TEM images at different magnifications of $\bar{{\mathrm{MoS}}_{2}}$/ml-G-2 after scratching the film from the quartz substrate (**a**–**c**). Image a shows a general view where the presence of MoS_2_ as darker particles can be seen embedded within the multilayer graphene film in lighter contrast. The inset of Panel c shows the FFT, and (**d**) shows the measurement of the distance between different planes.

Unfortunately, TEM images of the $\bar{{\mathrm{MoS}}_{2}}$/ml-G films cannot be obtained without detaching them from the quartz substrate. Accordingly, TEM images of the $\bar{{\mathrm{MoS}}_{2}}$/ml-G-2 could only be obtained after scratching debris of the $\bar{{\mathrm{MoS}}_{2}}$/ml-G film from the quartz substrate. Figure 4 provides a set of images of pieces of the $\bar{{\mathrm{MoS}}_{2}}$/ml-G film detached from the quartz substrate. Figure 4a shows a larger image of the $\bar{{\mathrm{MoS}}_{2}}$/ml-G film showing the presence of $\bar{{\mathrm{MoS}}_{2}}$ particles (darker particles) surrounded by graphene characterized by lighter contrast. Figure 4b,c focuses on $\bar{{\mathrm{MoS}}_{2}}$ particles. In Panel c, the presence of graphene layers (lighter contrast) wrapping the MoS_2_ particle is clearly observed. High resolution TEM measurements of the interlayer distance of the particles give a value of 0.62 nm, which agrees with the value for the 002 interplanar distance in MoS_2_, thus providing an additional confirmation of the preferential 002 orientation of the MoS_2_ particles determined by XRD for those samples with high MoS_2_ loading [13]. Fast Fourier transform (FFT) showed that the MoS_2_ particles were highly crystalline. Figure 4 shows three selected TEM images at different magnifications, as well as the measurement of the interplanar distance and the FFT taken from the image.

**Figure 5:**
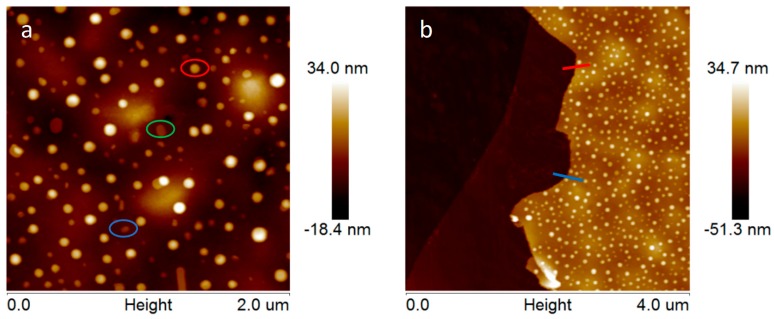

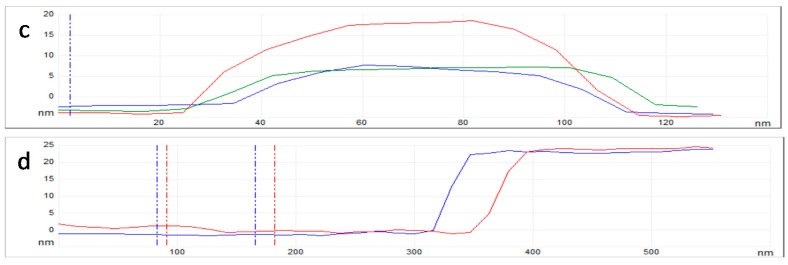
Top views of the AFM images at the central (**a**) and border (**b**) part of the $\bar{{\mathrm{MoS}}_{2}}$/ml-G-2 sample. (**c**) Shows the height and lateral dimensions of three representative MoS_2_ nanoparticles marked in the image with the corresponding red, green and blue color. (**d**) shows the section profile at two points of the edge of the film (red and blue) from which it can be determined that the thickness of the ml-G film is about 20 nm.

By using the AFM technique with subnanometric vertical resolution, the thickness of the $\bar{{\mathrm{MoS}}_{2}}$/ml-G-2 film and the nanoplatelet morphology of MoS_2_ particles grafted on G were confirmed. Figure 5 presents the measurement of the heights of three representative large MoS_2_ nanoplatelets with a lateral area of about 80 nm, showing that these particles are flat and thin, with heights between 5 and 15 nm. The thickness of the graphene film could also be measured at the edge of the scratch. It was determined that the average thickness of $\bar{{\mathrm{MoS}}_{2}}$/ml-G-2 film was about 20 nm (Figure 5d).

**Figure 6:**
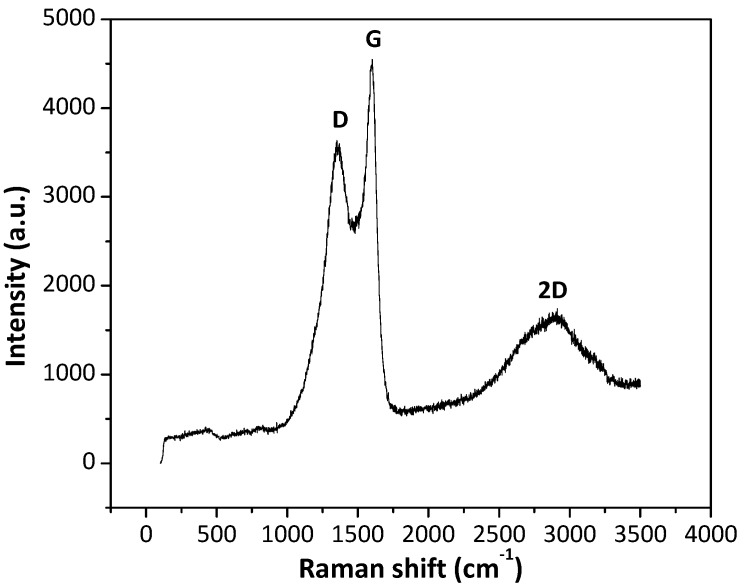
Raman spectra of the $\bar{{\mathrm{MoS}}_{2}}$/ml-G-2 sample.

Raman spectra of $\bar{{\mathrm{MoS}}_{2}}$/ml-G samples show the characteristic 2D, G and D peaks appearing at 2912, 1602 and 1367 cm^−1^, respectively, expected for defective G. As an example, Figure 6 shows the Raman spectra for $\bar{{\mathrm{MoS}}_{2}}$/ml-G-2 film. The intensity of the G vs. the D band (I_G_/I_D_) gives a quantitative indication of the density of defects of the G layers. In the present case, the I_G_/I_D_ value was about 1.26, which is higher than those I_G_/I_D_ values typically reported for reduced graphene oxide (rGO) samples, which are generally about 0.9 [14]. This indicates that G in the $\bar{{\mathrm{MoS}}_{2}}$/ml-G samples should have somewhat lower defect density than conventional rGO samples. No peaks due to the presence of MoS_2_ could be observed. MoS_2_ exhibits in Raman spectroscopy two characteristic A1g and E1g vibration modes at about 400 and 380 cm^−1^ [15] that could not be recorded in the present $\bar{{\mathrm{MoS}}_{2}}$/ml-G films. The low MoS_2_ loading together with the low intensity of their Raman bands is the most probable reason for the lack of MoS_2_ detection, as was already discussed when commenting on the XRD patterns.

**Figure 7:**
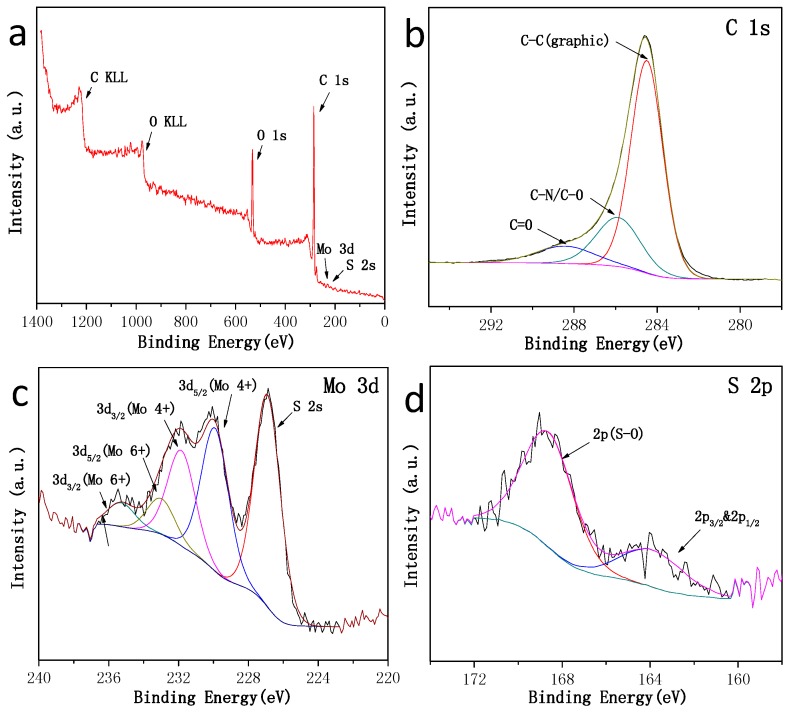
High-resolution XPS spectra of the $\bar{{\mathrm{MoS}}_{2}}$/ml-G-2 material. (**a**) Survey spectrum; (**b**) C 1s spectrum; (**c**) Mo 3d spectrum and S 2p; (**d**) other region of the S 2p spectrum. Note that Mo and the main S peaks appear in (**c**), where some components corresponding to Mo (6+) have been marked.

The chemical states of Mo, S and C in the $\bar{{\mathrm{MoS}}_{2}}$/ml-G sample were investigated by carrying out the X-ray photoelectron spectroscopy (XPS) measurements (Figure 7). The survey XPS spectrum of $\bar{{\mathrm{MoS}}_{2}}$/ml-G film shows the presence of the expected C, O, Mo and S elements, the latter two with very low intensity. In addition to the lower response factor of these two elements with respect to C and O, the low intensity of Mo and S peaks could indicate that $\bar{{\mathrm{MoS}}_{2}}$ particles are not well exposed to the external surface and that they are wrapped by G layers. The high resolution XPS spectra show that the C 1s peak can be resolved into three peaks centered at 284.5 (68.1%), 285.9 (10.2%) and 288.4 eV (21.7%), which could correspond to graphitic carbons, C–O/C–N and C=O, respectively. The Mo 3d spectrum spectra shows the existence of the Mo (4+) oxidation state (73.4%), as well as the Mo (6+) oxidation state (26.6%), the latter probably due to the formation of some MoO_3_ (about 20%) on the surface of the nanoparticles upon exposure to air [16]. It should be noted that the presence of some MoO_3_ only corresponds to the outermost surface of the sample probed by XPS and that elemental analysis by SEM confirms the MoS_2_ stoichiometry. The presence of a high proportion of MoS_2_ on the surface of the material is also confirmed by the observation of the corresponding S 2s peak at 226.5 eV binding energy near the Mo peak (Figure 7c) corresponding to about 80% of all the S atoms. The presence of some S–O band (18%) and a small amount of bridging S_2_^2−^ (4%) were also detected in a different binding energy value region by the corresponding S 2p at 164.0 and 163.2 eV (Figure 7d) [17].

### 2.2. Electrocatalytic Measurements

Square $\bar{{\mathrm{MoS}}_{2}}$/ml-G films supported on quartz of a surface of 2 × 2 cm^2^ resulting from the pyrolysis of alginate precursors were directly used as electrodes for H_2_ generation. Note that $\bar{{\mathrm{MoS}}_{2}}$/ml-G films are not coating the glassy carbon electrode or any other conductive substrate and that the electrical conductivity in $\bar{{\mathrm{MoS}}_{2}}$/ml-G derives from the intrinsic properties of the graphitic carbon forming the film. Previous reports in the literature have established that films’ defective Gs obtained by pyrolysis of natural polysaccharide on quartz substrates exhibit notable electrical conductivity, with surface resistivity values in the range of kΩ × □ [7,11]. The fact that no conductive electrode is needed in the case of $\bar{{\mathrm{MoS}}_{2}}$/ml-G is one important advantage derived from the preparation procedure and from the composition of the samples. 

**Figure 8:**
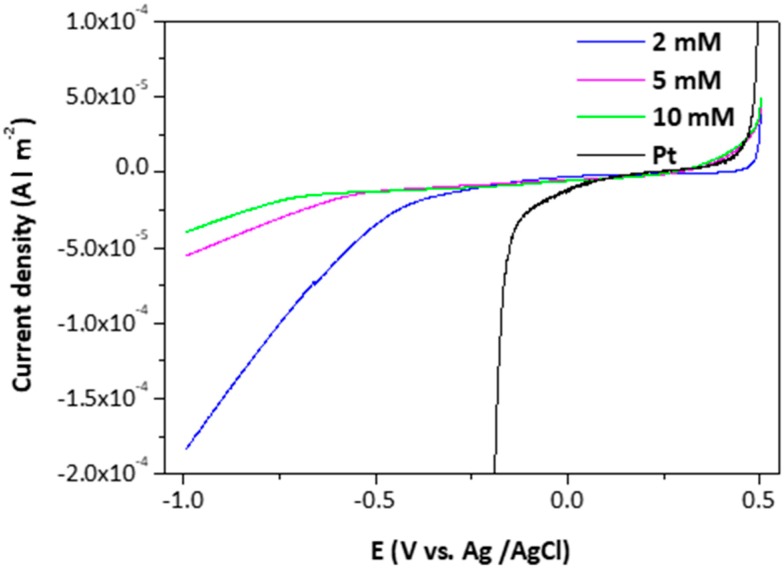
Polarization curves of the $\bar{{\mathrm{MoS}}_{2}}$/ml-G films and Pt nanoparticles for hydrogen evolution reaction (HER) activity.

Representative measurements of the electrocatalytic behavior of the $\bar{{\mathrm{MoS}}_{2}}$/ml-G films for H_2_ generation are presented in Figure 8, where the performance of $\bar{{\mathrm{MoS}}_{2}}$/ml-G films is compared to that of Pt nanoparticles deposited on glassy carbon. As can be seen there, differences in the onset for H_2_ generation and in the current density of the $\bar{{\mathrm{MoS}}_{2}}$/ml-G electrodes as a function of the concentration of (NH_4_)_2_MoS_4_ used in the preparation of the electrodes were observed, there being an optimal loading corresponding to (NH_4_)_2_MoS_4_ close to 2 mM. The observation of an optimal loading typically occurs when there are two opposite factors related to the amount of MoS_2_ influencing the electrocatalytic activity. We propose that these two factors are the catalytic activity of MoS_2_ for H_2_ evolution that should increase as the loading of MoS_2_ increases and the increase in the particle size of MoS_2_ with lesser density of defects that should disfavor the catalytic activity as MoS_2_ loading increases. It is known that the electrocatalytic activity of MoS_2_ derives from steps and defects on the nanoparticles [18,19], and these defects should be more abundant when the particle size is smaller, a fact that should occur at low MoS_2_ loadings. As mentioned earlier, SEM images clearly indicate that the particle size grows from 15–105 nm upon increasing (NH_4_)_2_MoS_4_ concentration. On the other hand, for low MoS_2_ loadings, the density of active sites in 2 × 2 cm^2^ should be low, resulting in low activity, as was the case of the $\bar{{\mathrm{MoS}}_{2}}$/ml-G film prepared using the 0.5 M (NH_4_)_2_MoS_4_ concentration. As a result, a compromise should be reached at an optimal MoS_2_ loading close to a 2 mM (NH_4_)_2_MoS_4_ concentration during the preparation of the $\bar{{\mathrm{MoS}}_{2}}$/ml-G films.

**Figure 9:**
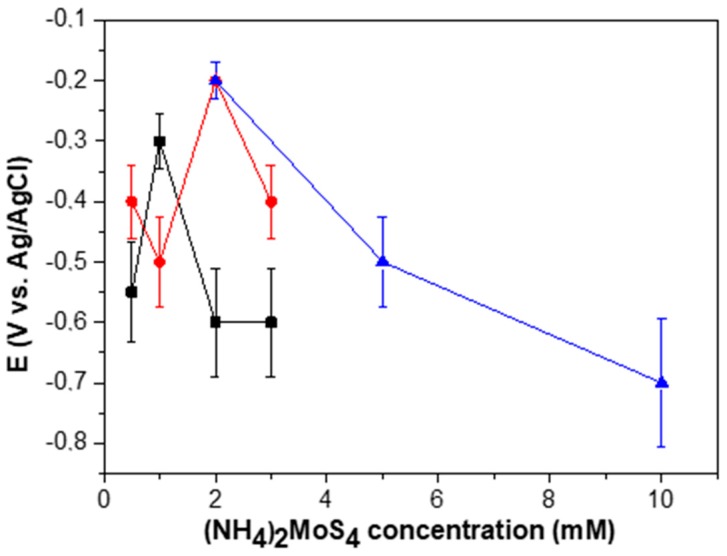
The electrocatalytic performance characterized by the onset potential for H_2_ evolution of three independent batches of samples (in different colors) as a function of the concentration of (NH_4_)_2_MoS_4_ precursor.

By performing a series of independent electrode preparations, it was observed that the electrocatalytic response of the $\bar{{\mathrm{MoS}}_{2}}$/ml-G films was not exactly reproducible from one batch to the other, there being a dispersion on the potential onset of H_2_ generation and the current density achieved at different potentials for the $\bar{{\mathrm{MoS}}_{2}}$/ml-G films as a function of the (NH_4_)_2_MoS_4_ concentration. Figure 9 presents data of three independent sets of $\bar{{\mathrm{MoS}}_{2}}$/ml-G film preparation showing the variation in the response of the electrodes. We suggest that this lack of complete reproducibility derives in a large extent from the difficulty to make electrical contacts on films of nanometric thickness and on the random growth of MoS_2_ particles during the pyrolysis, particularly in the low concentration range. In any case, whatever the reason, independent preparation of several series of $\bar{{\mathrm{MoS}}_{2}}$/ml-G films showed that the optimal values of the concentration were in the range between 1 and 2 mM (see Figure 9), for which an onset potential of −0.2/−0.3 V is consistently measured, with Tafel slopes of 180 mV/decade. Thus, the dispersion in the behavior of the electrodes, although existing, allows a degree of confidence on the performance of $\bar{{\mathrm{MoS}}_{2}}$/ml-G films. In the literature, an onset potential for MoS_2_ supported on reduced graphene oxide deposited on a conductive glassy carbon electrode of 100 mV with a rise of 41 mV/decade was reported on a 0.5 M H_2_SO_4_ aqueous solution [4]. Note, however, that although the electrolyte solutions in the reported data and the present study are the same, other conditions and, particularly, the absence of a conductive electrode and the use of flat quartz substrate as the electrode are remarkably different from those used in the literature [4].

Regarding stability, it was observed that $\bar{{\mathrm{MoS}}_{2}}$/ml-G films undergo easy peeling off from the quartz substrate upon a few electrocatalytic measurements. This reflects poor adherence of $\bar{{\mathrm{MoS}}_{2}}$/ml-G films to the quartz substrate.

## 3. Conclusions

In the present manuscript, it is reported that large surface area of films of $\bar{{\mathrm{MoS}}_{2}}$/ml-G on arbitrary, non-conductive substrates can be prepared in one step by pyrolysis at 900 °C under Ar of ammonium alginate films containing (NH_4_)_2_MoS_4_. During the pyrolytic process, two separate phases corresponding to graphitic carbon (multilayer graphene) and MoS_2_ develop spontaneously. MoS_2_ platelets exhibit a preferential 002 facet orientation, and they have affinity for graphene as deduced from the relative lateral surface area to height ratio, which is large. The $\bar{{\mathrm{MoS}}_{2}}$/ml-G films act as electrocatalysts for H_2_ generation without the need for any conductive electrode, exhibiting a potential onset between −0.2 and −0.3 V depending on the concentration of (NH_4_)_2_MoS_4_ used in the preparation, with certain variation of the electrocatalytic performance from batch to batch. Considering the simplicity of the one-step preparation procedure and the precursors, the present protocol is advantageous for the preparation of $\bar{{\mathrm{MoS}}_{2}}$/ml-G films as electrocatalysts in an easily scalable way.

## 4. Experimental Section

### 4.1. Synthesis of the Oriented $\bar{MoS_{2}}$/ml-G Films

Alginic acid (1200 mg) from Aldrich (St. Louis, MO, USA) was suspended in aqueous solutions containing different concentrations of (NH_4_)_2_MoS_4_ (0.5, 1, 2, 3, 5, 10 or 60 mM). Two milliliters of NH_4_OH solution (28–30% NH_3_ in water) were added to dissolve alginate acid completely. After 2 h under magnetic stirring at room temperature, the solutions were filtered through a syringe of a 0.45 µm pore size to remove insoluble impurities present in the commercial alginic acid. The films were cast on a previously cleaned quartz plate (2 × 2 cm^2^) by spin coating 500 µL of filtered ammonium alginate solution at 4000 rpm for 45 s. Once dried on a hot plate, the films were pyrolyzed under Ar flow (200 mL·min^−1^), increasing the temperature at a rate of 5 °C·min^−1^ up to 900 °C and a holding time of 1 h. After this time, the films were cooled down at room temperature also under Ar flow.

### 4.2. Characterization Techniques

XRD patterns were obtained by using a Philips X’Pert diffractometer (Philips, Amsterdam, The Netherlands) and copper radiation (CuK_a_ = 1.541178 Å). Raman spectra were recorded at ambient temperature with 514 nm laser excitation on a Renishaw In Via (New Mills, UK) Raman spectrometer equipped with a CCD detector. TEM images were recorded by using a Philips CM 300 FEG system with an operating voltage of 100 kV after scratching $\bar{{\mathrm{MoS}}_{2}}$/ml-G films with a cutter. AFM images were made with Multimode Nanoscope 3A equipment (Bruker, Billerica, MA, USA) working in tapping mode, using mica as the substrate. Field emission scanning electron microscopy (FESEM) images were taken with an ULTRA 55 ZEISS Oxford instrument (Pleasanton, CA, USA) and high-resolution transmission electron microscopy (HRTEM) images with a JEM 2100F JEOL 200-kV electronic microscope (JEOL, Akishima, Japan) employed to collect the morphology of the solid samples and element mapping of selected areas. XPS analyses were measured using a SPECS spectrometer with a MCD-9 detector exciting with a monochromatic Al (K = 1486.6 eV) X-ray source (SPECS, Berlin, Germany). Peak deconvolution fittings were performed using the CASA software (Version 2.3.14 dev3, RBD, Bend, OA, USA) using the C 1s peak at 284.4 eV as the binding energy reference.

### 4.3. Electrochemical Characterization 

Electrocatalytic measurements of $\bar{{\mathrm{MoS}}_{2}}$/ml-G electrodes were carried out using a potentiostat/galvanostat (VersaSTAT 3, Princeton Applied Research, Oak Ridge, TN, USA) with a standard three-electrode cell configuration. $\bar{{\mathrm{MoS}}_{2}}$/ml-G films were used as the working electrode. Ag/AgCl/KCl (3 M) and Pt wire were used as the reference and counter electrode, respectively. An aqueous solution of 0.5 M of H_2_SO_4_ was used as the electrolyte and was degassed using argon. Onset potentials were measured by extrapolating to zero current density the initial linear part of the V-J plot. The standard error of the measurement, based on three independent batches, was estimated to be 20%.

## Data Availability

Datasets are available from the authors upon request.
